# Towards a better understanding of preimplantation genetic screening for aneuploidy: insights from a virtual trial for women under the age of 40 when transferring embryos one at a time

**DOI:** 10.1186/s12958-017-0269-y

**Published:** 2017-06-30

**Authors:** Paul N. Scriven

**Affiliations:** Genetics Laboratories, 5th Floor Tower Wing, Guy’s Hospital, Great Maze Pond, London, SE1 9RT UK

**Keywords:** PGS, Aneuploidy, Cost-effectiveness, Diagnostic accuracy

## Abstract

**Background:**

The aim of this theoretical study is to explore the cost-effectiveness of aneuploidy screening in a UK setting for every woman aged under the age of 40 years when fresh and vitrified-warmed embryos are transferred one at a time in a first full cycle of assisted conception.

**Methods:**

It is envisaged that a 24-chromosome genetic test for aneuploidy could be used to exclude embryos with an abnormal test result from transfer, or used only to rank embryos with the highest potential to be viable; the effect on cumulative outcome is assessed. The cost associated with one additional live birth event and one clinical miscarriage avoided is estimated, and the time taken to complete a cycle considered. The numbers of individual woman for whom testing is likely to be beneficial or detrimental is also evaluated.

**Results:**

Adding aneuploidy screening to a first treatment cycle is unlikely to result in a higher chance of a live birth event, and can be detrimental for some women. Premature termination of a clinical trial is likely to be biased in favour of genetic testing. Testing is likely to be an expensive way of reducing the chance of clinical miscarriage and shortening treatment time without a substantial reduction in the cost of testing, and is likely to benefit a minority of women. Selecting out embryos is likely to reduce the treatment time for women whether or not they have a baby, whilst ranking embryos only to reduce the time for those that have a child and not for those who need another stimulated cycle.

**Conclusions:**

Adding aneuploidy screening to IVF treatment for women under the age of 40 years is unlikely to be beneficial for most women. To achieve an unbiased assessment of the cost-effectiveness of genetic testing for aneuploidy, clinical trials need to take account of women who still have embryos available for transfer at the end of the study period. Specifying the proportions of women for whom testing is likely to be beneficial and detrimental may help better inform couples who might be considering adding aneuploidy screening to their treatment cycle.

**Electronic supplementary material:**

The online version of this article (doi:10.1186/s12958-017-0269-y) contains supplementary material, which is available to authorized users.

## Background

Multiple birth is recognised to be an important risk to the health and welfare of children born after in vitro fertilisation (IVF), and can be effectively reduced by transferring only one embryo to those women who are most at risk of having twins [1]. The current National Institute for Health and Care Excellence (NICE) guideline covering diagnosing and treating fertility problems in the UK recommends state-funding and single embryo transfer (fresh or cryopreserved) in the first full IVF cycle for women under 37 years, and if there are one or more top-quality embryos for women aged 37 to 39 years [2]. UK state-funding is not available currently for preimplantation genetic screening (PGS).

Current genetic testing techniques for chromosome aneuploidy can test for every chromosome [3]. Selecting embryos with the highest potential for implantation offers the potential to transfer one embryo at a time in the fewest possible number of transfer procedures to optimise a woman’s chance of achieving a healthy singleton live birth event and reduce the risk of miscarriage due to chromosome aneuploidy. Appropriately powered, well-designed, peer-reviewed randomised control trials, with a live birth outcome measure which goes on to report on child health, are recommended to be the gold standard for evidenced-based IVF medicine [4]. Although, others have argued for a more pragmatic approach to circumnavigate protracted delay in introducing the highest quality treatment for patients [5].

Outcome measures which incorporate fresh as well as cryopreserved embryo transfer (cumulative rates) rather than success rates based on only fresh transfer is recognised to be more appropriate for decision making regarding the efficacy of treatment and cost [6]. However, a prospective intention-to-treat embryo selection study is likely to be costly and take several years to complete, and difficult to justify if it is expected that the cumulative live birth rate (CLBR) with testing will be inferior due to an imperfect genetic test which incorrectly excludes viable embryos [7]. An inferior CLBR can be avoided if testing is used only to determine the order in which embryos will be transferred [8].

An aneuploidy screening trial using virtual women and embryos is an idealistic way of investigating the usefulness of different approaches and the cost-effectiveness of testing embryos, free from the principle of equipoise and the constraints of cost and time. The aim of the study presented here is to explore the cost-effectiveness of aneuploidy screening for every woman aged under the age of 40 years when fresh and vitrified-warmed embryos are transferred one at a time in a first full cycle of IVF, comparing selecting out with ranking-only, and with different trial end points.

## Methods

### Study outline

Suitable fresh and vitrified-warmed embryos from the first stimulated cycle were envisaged to be transferred one at a time until a first live birth event was achieved or there were no more embryos available (a full cycle). A priori it was assumed that, with effective cryopreservation and a test for 23 chromosome pairs with high accuracy, the cumulative live birth rate was likely to be similar with or without genetic testing; however, testing would be expected to result in fewer clinical miscarriages with fewer warmed cycles required overall [7]. On an intention-to-treat basis, the primary outcome measures were the cumulative clinical miscarriage and live birth rates per woman, and the time taken to complete a full cycle with and without a live birth event. A sample size of 980 (rounded up to 1000) virtual women, without and then with genetic testing that excludes embryos, was calculated to detect a 40% reduction in cumulative clinical miscarriage rate per cycle, from 6% without testing to 3.6% (single sided, 80% power, alpha 5%) [7, 9]. Two strategies with genetic testing were compared to not testing; where embryos with an abnormal test result were:

i) Excluded from transfer – PGS1
ii) Available for transfer but ranked below those with a normal test result – PGS2

A final optimized comparison was made with a hypothetical ideal test (PGS3), which enabled only viable diploid embryos to be selected for transfer in the fresh cycle (100% diagnostic accuracy, no clinical miscarriages and no vitrified-warmed cycles). Not-testing, PGS1, PGS2 and PGS3 cycle data are provided as additional material (see Additional file 1: Tables S1–S4).

Embryos were transferred in the same order for each comparison; however, for PGS, embryos with a euploid test result were transferred before those with an aneuploid result. In the first instance it was assumed that every woman would complete her treatment cycle, and then the same comparisons were made assuming that the trial ended after two years, and a second premature termination scenario where women discontinued treatment after a total of one fresh transfer and three vitrified-warmed transfers (“frozen” embryo transfer cycle, FET) without a live birth event.

Cycle turnaround times for individual women were estimated assuming:

i) Stimulated cycle with or without a fresh transfer – 1 month
ii) Failed transfer attempt interval – 3 months
iii) Clinical miscarriage interval – 6 months
iv) Live birth event – 9 months

### Study probabilities and population characteristics

Table 1 (pooled women) and Additional file 1: Table S5 (stratified for maternal age) show the study population characteristics. Reproductive outcome and diagnostic accuracy probabilities were obtained from a published prospective non-selection study, where embryo transfer occurred without using the results from the genetic test [10], and as described previously [7]. Aneuploid and euploid genetic test result probabilities were based on a large microarray chromosome study [11], respectively: 0.28949 and 0.71052 (<35 years), 0.37415 and 0.62585 (35–37 years), 0.50258 and 0.49742 (38–39 years). The numbers of euploid and aneuploid results in an individual embryo cohort were decided using a randomly generated probability and probability ranges calculated using binomial theorem. For example, <35 years and four embryos (n euploid, aneuploid): 0–0.25486 (4e,0a); >0.25486 < 0.67019 (3e,1a); >0.67019 < 0.92403 (2e,2a); >0.92403 < 0.99298 (1e,3a); >0.99298–1 (0e,4a). The order for transfer without genetic testing was decided by random assignment within each embryo cohort. A 94% warming survival rate was assumed from a published study [12]. Probability ranges were constructed for different possible outcomes, and any given event was decided using a randomly generated probability. For example, to decide the outcome of thawing a cryopreserved embryo, a randomly generated probability between 0 and 0.94 indicated survival and >0.94 indicated failure. To decide the outcome of a transferred embryo, a probability between 0 and 0.4361 {(55 + 3)/133, calculated from [10]} decided that an embryo with a euploid test result would result in a clinical pregnancy, and >0.4361 in implantation failure. In the event of achieving a clinical pregnancy, a probability between 0 and 0.0517 {3/(3 + 55), calculated from [10]} decided that the pregnancy would miscarry and >0.0517 that the pregnancy would survive to term. The corresponding probability thresholds for embryos with an aneuploid test result were 0.0606 {(59 + 5)-(3 + 55)/(232–133)} and 0.3333 {(5–3)/((59 + 5)-(55 + 3))}, calculated from [10]. Live birth events from an embryo with an aneuploid test result (predicted not to result in a live birth event) represented false positives.

**Table 1:** Hypothetical population characteristics and diagnostic accuracy measures

Virtual women (cycles) started (N)	1000
Age (y), median (range)	33 (22–39)
Cycles abandoned (n); %	15; 1.5
Oocyte retrievals (n); %	985; 98.5
Insemination by ICSI (n); %	660; 67.0
Insemination by IVF (n); %	325, 33.0
Cycles with embryos suitable for transfer or testing (n); %	870; 87.0
Embryos suitable for transfer or testing (N); median (range)	4476; 5 (1–14)
Diploid (n); %, median (range)	3073; 68.7; 3 (0–11)
Aneuploid (n); %, median (range)	1403; 31.3; 1 (0–11)
Embryos transferred, without testing, to achieve a first live birth event (n)	2204
^a^Positive predictive value [TP/(TP + FP)]; %	650/683; 95.2
^b^Negative predictive value [TN/(TN + FN)]; %	615/1521; 40.4
Live birth events (n)/fresh transfers (n); %	244/870; 28.0
Miscarriages (n)/fresh clinical pregnancies (n); %	27/271; 10.0

^a^Test perspective without acting on the test result, the proportion of embryos transferred with an abnormal (aneuploid) test result with a correct prediction of no live birth event; ^b^the proportion with a normal (euploid) test result that correctly predict a live birth event

### Costs

Assisted conception costs were based on the current self-funding price list for a single UK clinic [13]. The cost for aneuploidy screening was based on the median current self-funding price of four UK providers [14] (Table 2). It was assumed that eggs were fertilized using ICSI only if it was indicated for treatment and not a prerequisite for genetic testing.

**Table 2:** Costs

Procedure	Cost £ (range)
An in vitro fertilization (IVF) cycle^a^	3300
Intracytoplasmic sperm injection (ICSI)	900
Stimulation drugs	900
Embryo freezing and storage	800
Frozen embryo transfer (FET)^b^	1400
FET drugs	150
Aneuploidy screening (PGS)^c^	2965 (2950–3000)

^a^Assumed to exclude the cost for medication, and to include medical and nurse appointments, scans, egg collection, blastocyst culture, embryo transfer, and a pregnancy scan or a follow up consultation with the doctor. ^b^Assumed to exclude the cost for medication, and to include consultations, scans and follow-up. ^c^Assumed to include the cost for biopsy and testing one or more embryos

Cost was assessed using the incremental cost-effectiveness ratio (ICER) [15], the difference in cost for one clinical miscarriage avoided or one additional live birth event achieved. An additional calculation was made to estimate the genetic test cost required so that the overall cost with PGS was not more than the overall cost of IVF without genetic testing.

### Statistics

The odds ratio and 95% confidence interval was used to measure the effect of genetic testing on cumulative outcomes [16], and controlled for maternal age (<38 years vs 38–39 years) using logistic regression [17]. A *P*-value of less than 0.05 was considered to be statistically significant (*).

## Results

### Intention-to-treat outcomes

Table 3 shows the number of clinical miscarriages and live birth events following a fresh transfer and each vitrified-warmed (FET) attempt, and the cumulative rates without premature trial termination.

**Table 3:** Hypothetical intention-to-treat cumulative outcomes

1000 virtual women started	Fresh	FET1	FET2	FET3	FET4	FET5	FET6	FET7	FET8	FET9
Not-tested cycles (n)	1000	596	362	199	102	48	25	13	2	1
Not-tested transfer attempts (n)	870	593	355	197	100	48	25	13	2	1
Clinical miscarriages (n)	27	14	6	8	0	0	1	0	0	0
CCMR (%)	2.7	4.1	4.7	5.5	5.5	5.5	5.6	5.6	5.6	5.6
Live birth events (n)	244	170	121	59	26	14	6	7	0	1
CLBR (%)	24.4	41.4	53.5	59.4	62.0	63.4	64.0	64.7	64.7	64.8
PGS1 cycles (n)	1000	440	189	66	21	10	3	1	0	0
PGS1 transfer attempts (n)	841	434	186	64	21	10	3	1	0	0
Clinical miscarriages (n)	27	7	2	0	1	0	0	0	0	0
CCMR (%)	2.7	3.4	3.6	3.6	3.7	3.7	3.7	3.7	3.7	3.7
Live birth events (n)	335	174	78	30	7	6	2	1	0	0
CLBR (%)	33.5	50.9	58.7	61.7	62.4	63.0	63.2	63.3	63.3	63.3
PGS2 cycles (n)	1000	502	261	140	59	29	14	6	1	0
PGS2 transfer attempts (n)	870	500	255	138	58	28	14	6	1	0
Clinical miscarriages (n)	27	10	2	5	1	0	0	1	0	0
CCMR (%)	2.7	3.7	3.9	4.4	4.5	4.5	4.5	4.6	4.6	4.6
Live birth events (n)	336^a^	176	85	35	7	6	2	1	0	0
CLBR (%)	33.6	51.2	59.7	63.2	63.9	64.5	64.7	64.8	64.8	64.8
PGS3 cycles (n)	1000	0	0	0	0	0	0	0	0	0
PGS3 transfer attempts (n)	652	0	0	0	0	0	0	0	0	0
Clinical miscarriages (n)	0	0	0	0	0	0	0	0	0	0
CCMR (%)	0	0	0	0	0	0	0	0	0	0
Live birth events (n)	652	0	0	0	0	0	0	0	0	0
CLBR (%)	65.2	65.2	65.2	65.2	65.2	65.2	65.2	65.2	65.2	65.2

^a^Includes a live birth event from a cohort comprising one embryo which had a false abnormal test result

**Fig. 1:**
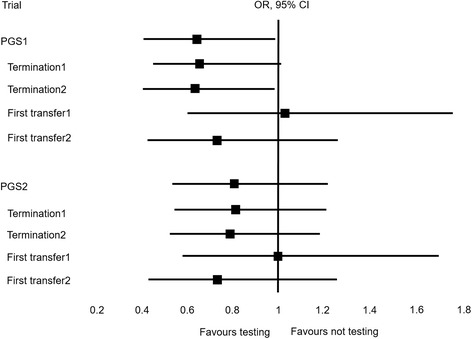
The hypothetical effect of genetic testing on the chance of clinical miscarriage. Legend: Termination1 and 2; virtual trial discontinued after two years and four transfer attempts respectively. First transfer1 and 2; rates calculated using the numbers of first transfer attempts and clinical pregnancies respectively

**Fig. 2:**
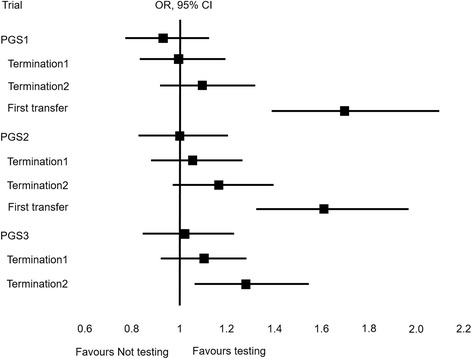
The hypothetical effect of genetic testing on the chance of a live birth event

The effect of testing on the cumulative clinical miscarriage rate (CCMR) and the cumulative live birth rate (CLBR) for 1000 women started is shown in Figs. 1 and 2 respectively (see also Additional file 1: Table S6). Completing a treatment cycle, a woman had a 0.648 times (*P* = 0.0451*) and 0.813 times (*P* = 0.3094; a sample size of 5984 women would be required for *P* < 0.05) higher chance of clinical miscarriage with PGS1 (embryo exclusion) and PGS2 (embryo ranking) respectively. A woman had a 0.937 times (*P* = 0.4846; 12,654 women would be required for *P* < 0.05), 1 times (*P* = 1) and 1.018 times (*P* = 0.8513; 175,816 women would be required for *P* < 0.05) higher chance of a live birth event with PGS1, PGS2 and PGS3 (ideal test) respectively. Maternal age, by design, was not a confounding factor; however, it was an important independent predictor of live birth and a younger woman (<38y) had a 2.6 times (*P* < 0.0001) higher chance of a live birth event than an older woman (38-39y). With small numbers, a younger woman had a 0.683 times (*P* = 0.2028, PGS1) and 0.647 times (*P* = 0.1231, PGS2) higher chance of a clinical miscarriage than older women (see Additional file 1: Tables S7–S9).

Considering only the first transfer attempt and testing with PGS1 and PGS2 respectively, a woman had a 1.699 times (*P* < 0.0001) and 1.614 times (*P* < 0.0001) higher chance of a live birth, and a 1 times (*P* = 1) and 1.036 times (*P* = 0.8993) higher chance of a clinical miscarriage. The number of women with a miscarriage following the first transfer attempt was the same with and without testing, but there were more women with a clinical pregnancy following testing. Considering only those women who achieved a clinical pregnancy, women following testing with PGS1 and PGS2 respectively had a 0.728 times (*P* = 0.2643) and 0.726 times (*P* = 0.2598) higher chance of clinical miscarriage (see also Additional file 1: Table S10).

Without testing, 20 (2%) women had embryos remaining and no live birth event after 25 months (Termination 1); compared to 5 (0.5%) with PGS1, 7 (0.7%) with PGS2 and 0 (0%) for PGS3. Terminating the trial after a total of one fresh and three vitrified-warmed transfer attempts (Termination 2), without testing, 102 (10.2%) women still had embryos available for transfer, compared to 22 (2.2%) with PGS1, 63 (6.2%) with PGS2 and 0 (0%) for PGS3. Premature termination reduced the number of clinical miscarriage events in the not-tested group from 56 to 55 events (−1.8% for Termination 1 and 2). Premature termination had a larger and disproportionate effect on the number of live birth events without genetic testing: 648 women vs 629 (−2.9% for Termination 1) and 594 (−8.3% for Termination 2). The effect of premature trial termination is shown in Figs. 1 and 2 (see also Additional file 1: Table S6).

**Fig. 3:**
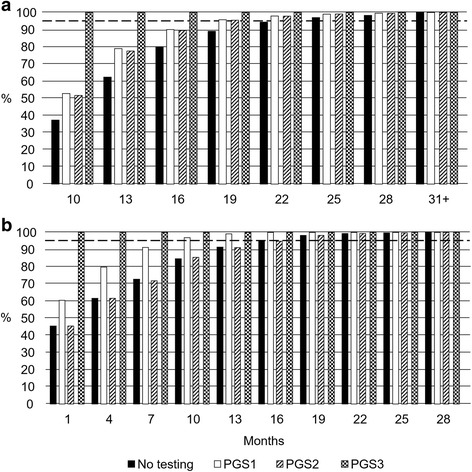
Time to complete a hypothetical full cycle without premature termination of the virtual trial Legend: Cumulative rates for women where a live birth event was achieved (**a**), and where a live birth event was not achieved (**b**)

Without testing, the total treatment time ranged from 1 to 43 months. With testing, the total treatment time ranged from 1 to 31 months for PGS1 and PGS2, and from 1 to 10 months for PGS3. Without genetic testing, 95% of women who had a live birth event achieved this within 25 months (Fig. 3a), compared to 19 months with PGS1 and PGS2, and 10 months for PGS3. Without genetic testing, 95% of women who did not achieve a live birth event completed their cycle within 16 months (Fig. 3b), compare to 10 months with PGS1, 16 months for PGS2 and 1 month with PGS3.

### Cost-effectiveness

Table 4 (see also Additional file 1: Tables S11–S17) shows a summary of the analyses comparing genetic testing with not testing where every woman completed a cycle without prematurely terminating treatment. When embryos with an abnormal test result were excluded from transfer (PGS1), the cost of avoiding one miscarriage was £81,013 [(£9,086,650 - £7,547,400)/(56–37)]; however, fewer women had a live birth event (633 with testing versus 648 without testing) due to the exclusion of viable embryos with false positive test results, and was more expensive for 73.3% (733/1000) of women. With genetic testing, 19 (1.9%) women avoided a miscarriage, of which it was less expensive for 9 (0.9%) and more expensive for 10 (1%). Testing was detrimental for 1 (0.1%) women due to a live birth event associated with an embryo with a false positive test result, which occurred in an earlier transfer attempt when not acting on the test results, than a miscarriage associated with a later transfer attempt of embryos with euploid results following genetic testing. The time to complete the cycle was reduced for 414 (41.4%) women (median 3 months, range 3–27 months), of which it was less expensive for 145 (14.5%) and more expensive for 269 (26.9%). The time was increased for 7 (0.7%) women (median 3 months, range 3–12 months) due to the exclusion of viable embryos with a false positive test result.

**Table 4:** Cost-effectiveness summary: genetic testing versus not testing for 1000 virtual women

Group	Outcome	n	FET cost (£)	PGS cost (£)	Total cycle cost (£)	ICER (£)	More effective (n)			No effect (n)			Less effective (n)		
							Less expensive	Same expense	More expensive	Less expensive	Same expense	More expensive	Less expensive	Same expense	More expensive
No testing	Miscarriage	56	1550	-	7,547,400	-	-	-	-	-	-	-	-	-	-
	Live birth	648				-	-	-	-	-	-	-	-	-	-
PGS1	Miscarriage	37	1550	2965	9,086,650	81,013	9	0	10	144	114^a^	722	0	0	1^b^
	Live birth	633				N/A	0	0	0	152	114^a^	719	1^c^	0	14^c^
	Time						145	0	269	8	114^a^	457	0	0	7^c^
PGS2	Miscarriage	46	1550	2965	9,606,150	205,875	5	0	5	80	114^a^	795	0	0	1^b^
	Live birth	648				N/A	0	0	0	85	114^a^	801	0	0	0
	Time						77	0	176	8	114^a^	604	0	0	21^b,c,d^
PGS3	Miscarriage	0	1550	2965	7,955,150	7281	38	0	16	332	114^a^	500	0	0	0
	Live birth	652				101,938	4	0	0	366	114^a^	516	0	0	0
	Time						359	0	235	9	114^a^	281	2^e^	0	0

^a^Women with no embryos suitable for transfer or testing. ^b^PGS was less effective for miscarriage in one cycle due a live birth event associated with an embryo with a false positive test result, which occurred in an earlier transfer attempt when not acting on the test results, than a miscarriage associated with a later transfer attempt of embryos with euploid results following genetic testing. ^c^PGS less effective due to exclusion of false positives. ^d^PGS less effective due to the transfer order and fewer warming failures. ^e^PGS less effective for time due to a live birth event in the fresh transfer of an embryo following testing that did not survive warming in a later transfer attempt without testing

Genetic testing to rank embryos for transfer (PGS2) avoided fewer miscarriages (10 vs 19 with PGS1, and detrimental for 1 woman) and was more expensive than exclusion; however, testing was as effective as not testing for live birth. The ideal genetic test (PGS3) was the least expensive scenario, and 4 (0.4%) more women had a live birth event following testing, and was detrimental to none with respect to miscarriage. A reduction in the genetic testing cost from £2965 to £1195 (PGS1, −60%), £598 (PGS2, −80%) and £2496 (PGS3, −16%) was required to make the overall cost with genetic testing no more expensive than not testing.

A sub-group analysis for PGS1 vs not-testing included the 830 women who had more than one embryo available for transfer or testing (see also Additional file 1: Table S18). Following testing, there were fewer live birth events (625 vs 639) and fewer clinical miscarriages (36 vs 55); the cost of avoiding one clinical miscarriage was £74,771. Testing to avoid miscarriage was more effective and less expensive for 9 (1.1%) women. Testing was more expensive for 82.5% of women, and the time to complete the cycle was reduced for 49.8% of women. A reduction in the genetic testing cost to £1253 (−58%) was required to achieve parity of overall cost.

## Discussion

This virtual trial was conducted to provide insight into the cost-effectiveness of incorporating PGS into the first IVF treatment attempt for every woman under the age of 40 years, transferring embryos one at a time. It was envisaged that a 24-chromosome genetic test for aneuploidy could be used to exclude embryos with an abnormal test result from transfer (PGS1), or be used only to rank embryos with the highest potential to be viable (PGS2), with the effect on outcome of prematurely discontinuing treatment also assessed. These approaches were compared with treatment without testing. A hypothetical ideal test was also considered, which could ensure that only a viable embryo, if available, was transferred first (PGS3), with no risk of miscarriage.

Testing, by design, was effective to reduce the occurrence of clinical miscarriage. This was based on a prospective non-selection study [10] where the clinical miscarriage rate per clinical pregnancy was calculated to be 8.5% (5/59) without genetic testing and 5.1% (3/59) following aneuploidy screening [RR 0.6 (0.15–2.397) *P* = 0.4639]. The numbers are small and the confidence interval is wide and caution regarding the effect of testing is advised. In the virtual trial the miscarriage rates without testing were 9.6% (24/251) for younger women (<38 years) and 15% (3/20) for older women (38–39 years) (see also Additional file 1: Table S5). A UK cross-section audit including fresh and freeze-thawed cycles reported to the HFEA for clinics indicated similar rates for younger and older women of 9.0% (1128/12,524) and 15.3% (391/2558) (see also Additional file 1: Table S19). Excluding embryos (PGS1) was shown to be more effective and less expensive than ranking (PGS2); however, relatively few individual women were likely to benefit, whether on an intention-to-treat basis or when more than one embryo was available for testing. Testing did not result in a higher chance of a live birth event, and PGS1 was less effective than PGS2 due to the exclusion from transfer of viable embryos with incorrect abnormal test results (false positives).

Prematurely terminating the trial resulted in a disproportionate exclusion of women without testing who still had embryos available, which substantially reduced the deficit or resulted in an excess of live birth events following testing, with only a marginal effect on the number of clinical miscarriages avoided. Conclusions regarding the effectiveness of PGS for live birth based on clinical trials which include only the first transfer attempt, or that do not take into account women with surplus cryopreserved embryos, are therefore likely to be biased in favour of testing.

The author is aware of one published randomized controlled trial, for older women aged 38 to 41 years, which has attempted to estimate cumulative outcome measures [18]. After excluding poor prognosis patients, the primary outcome measure was the delivery (live birth event) rate for the first transfer attempt, which should be expected to favour testing [7]. The study reported a significantly higher live birth rate in the tested group: 52.9% (36/68) vs 24.2% (23/95) [OR 3.522 (1.804–6.873), *P* = 0.0002]. Adding live births from cryopreserved embryo transfers during the 6 months following the study recruitment period, the cumulative delivery rate in the tested group was reported to be 37.0% (37/100) vs 33.3% (35/105) [OR 1.175 (0.662–2.085), *P* = 0.5285]. It is not clear how many women after this period who had not achieved a live birth event still had cryopreserved embryos available. The cumulative miscarriage rate was 1.0% (1/100) with testing vs 20.0% (21/105) without testing; per 100 women, the cost of avoiding one miscarriage can be estimated to be €13,648 [(€1,075,273–€815,965)/(20–1)], with 19 (19%) women potentially benefiting from testing. Including all the embryos available from a stimulated cycle, it would seem that a woman in this age group is unlikely to increase her chance of having a baby, and around 1 in 5 of good prognosis patients is likely to avoid miscarriage by adding PGS to their treatment. Including fresh and cryopreserved embryo transfers, the authors reported a clinical pregnancy miscarriage rate of 36.2% (21/58) without testing vs 2.6% (1/38) with testing. A UK cross-section audit for older women (38 to 42 years) including fresh and freeze-thawed cycles reported to the HFEA for clinics with PGS activity indicated corresponding rates of 19.1% (859/4508) and 11.8% (14/119) (see also Additional file 1: Table S19). Consequently, the beneficial effect of testing on miscarriage indicated by the trial may be optimistic.

The treatment time, with or without a live birth event, was shortened for PGS1. The treatment time for women following PGS2 who had a baby was shortened, but it was not shortened for women who did not have a baby. Therefore, PGS2 would not enable women who needed more than one stimulated cycle to start the next cycle more quickly. This approach may also be unattractive to women because they would need to accept the possibility of transferring embryos with an abnormal test result to complete their cycle, which may also have some ethical hazard. However, it has been argued that the predictive value of genetic testing, even at the blastocyst stage, is too low for clinical use [19].

Using the hypothetical ideal genetic test (PGS3), testing was more effective for a live birth event than not testing, and the risk of a clinical miscarriage was eliminated. A woman’s cumulative live birth rate with PGS3 was superior to not testing because only fresh embryos were transferred, which avoided the attrition associated with a subsequent vitrified-warmed cycle [7]. A limitation of these hypothetical studies is the assumption that the live birth potential is the same for a fresh and warmed embryo transfer. A recent report of a randomized controlled trial [20], which compared fresh and vitrified-warmed embryo transfer following PGS, did not find a statistically significant difference in the number of live births per fresh (52%, 13/25) and warmed-vitrified (64%, 16/25) single embryo transfer, although the latter was greater with small numbers.

This virtual trial was based on a prospective non-selection study designed to assess diagnostic accuracy [10], where the value of an aneuploid test result to predict non-viability was high (96%). This may not be realistic if trophectoderm mosaicism is more common than recently appreciated [21]. However, unrealistically high diagnostic accuracy may be expected to favour testing and therefore testing may be more less-beneficial than indicated by this virtual trial. Using a different approach, another hypothetical trial also demonstrated greater superiority of not-testing over testing in terms of the cumulative live birth rate [22].

The implantation rate (44%) [10] of an embryo with a euploid test result used in this virtual trial might be considered to be modest. A higher implantation rate would not be expected to make genetic testing materially more effective than not testing for live birth over a full cycle (testing does not create normal embryos); however, it might be expected to reduce the number of warmed embryo transfer attempts and the cost of testing [7], and testing may therefore be less expensive than indicated by this virtual trial.

Every transferable embryo in this hypothetical study is assumed to have the same potential for implantation, differentiated only for those that are diploid or aneuploid. Augmenting selection using morphology criteria is outside the scope of this study; however, a mitigating effect on the number of warmed embryo transfers and cost might be expected.

## Conclusion

In the context of single embryo transfer for women under the age of 40 years, adding PGS universally to a first treatment cycle is likely to be an expensive way of reducing the risk of clinical miscarriage and shortening treatment time without a substantial reduction in the cost of genetic testing. PGS, whether by selecting out potentially aneuploid embryos or merely ranking potentially diploid embryos to be transferred first, is not expected to be superior for live birth when every available embryo is taken into account. A clinical trial which does not include all available embryos from a stimulation, or is terminated prematurely, is likely to be biased in favour of PGS. Selecting out embryos is likely to reduce the treatment time for women whether or not they have a baby, whilst ranking embryos will only reduce the time for those that have a child and not for those who need another stimulated cycle. Whether on an intention-to-treat basis or when more than one embryo is available for testing, a minority of women are likely to benefit, and it could be detrimental for some; presenting the likelihoods of both might help better inform couples considering adding aneuploidy screening to their IVF treatment.

## Additional file

Additional file 1:
**Table S1.** Dataset - Not tested. **Table S2.** Dataset - PGS1 (exclusion). **Table S3.** Dataset - PGS2 (ranking). **Table S4.** Dataset - PGS3 (ideal). **Table S5.** Population characteristics and diagnostic accuracy measures stratified for maternal age. **Table S6.** Effect of testing summary. **Table S7.** Logistic regression - PGS1. **Table S8.** Logistic regression - PGS2. **Table S9.** Logistic regression - PGS3. **Table S10.** Effect of testing - first transfer attempt. **Table S11.** Costing summary. **Table S12.** Cost-effectiveness analysis - PGS1 vs not testing - PGS £2,965. **Table S13.** Cost-effectiveness analysis - PGS1 vs not testing - PGS £1,195 (overall cost parity). **Table S14.** Cost-effective analysis - PGS2 vs not testing - PGS £2,965. **Table S15.** Cost-effective analysis - PGS2 vs not testing - PGS £598 (overall cost parity). **Table S16.** Cost-effective analysis - PGS3 vs not testing - PGS £2,965. **Table S17.** Cost-effective analysis - PGS3 vs not testing - PGS £2,496 (overall cost parity). **Table S18.** Cost-effectiveness analysis - PGS1 vs not testing - PGS £2,965, 2 or more embryos. **Table S19.** HFEA UK data collection for 16 clinics with PGS activity the period July 2011 to June 2014. (XLSX 2.77 mb)

## Abbreviations

CCMR: Cumulative clinical miscarriage rate
CLBR: Cumulative live birth rate
FET: Frozen embryo transfer
FN: False negative
FP: False positive
ICER: Incremental cost-effectiveness ratio
ICSI: Intracytoplasmic sperm injection
IVF: In vitro fertilization
NICE: National Institute for Health and Care Excellence
NPV: Negative predictive value
PGS: Preimplantation genetic screening
PPV: Positive predictive value
TN: True negative
TP: True positive
